# Correlation Between Tic Disorders and Serum 25-Hydroxyvitamin D Levels in Chinese Children

**DOI:** 10.3389/fped.2022.833371

**Published:** 2022-05-09

**Authors:** Simei Wang, Quanmei Xu, Anqi Wang, Fang Yuan, Xiaona Luo, Yilin Wang, Miao Guo, Yuanfeng Zhang, Wenjing Zhang, Xiaobing Ji, Yun Ren, Yucai Chen

**Affiliations:** Department of Neurology, Shanghai Children’s Hospital, Shanghai Jiao Tong University, Shanghai, China

**Keywords:** tic disorder, vitamin, children, 25-hydroxyvitamin D, deficiency

## Abstract

**Objective:**

To explore the correlation between serum 25-hydroxyvitamin D levels and tic disorders (TDs) in Chinese children.

**Methods:**

We selected 2960 children with TD and 2665 healthy controls, aged 5–14 years, from the Department of Neurology of the Shanghai Children’s Hospital. Serum 25-hydroxyvitamin D levels and degrees of vitamin D deficiency were compared between patients with TD and healthy children.

**Results:**

The mean serum 25-hydroxyvitamin D level in the TD group was significantly lower than that in the control group (*P* < 0.001). The proportion of patients with 25-hydroxyvitamin D deficiency in the TD group was significantly higher than that in the control group. However, there was no correlation between 25-hydroxyvitamin D deficiency and the severity of TD. In addition, for age-wise comparison, mean levels of 25-hydroxyvitamin D and its deficiency in the TD group were the most significant in children over 9 years of age.

**Conclusion:**

There is a correlation between 25-hydroxyvitamin D deficiency and TD in Chinese children, but not between 25-hydroxyvitamin D deficiency and the severity of TD. There was a correlation between age and deficiency of 25-hydroxyvitamin D; this deficiency was most pronounced among those over the age of 9 years.

## Introduction

Tic disorders (TDs) are chronic neuropsychiatric disorders that develop in childhood and affect up to 1% of the world’s population (1); they are characterised by a variety of motor or vocal tics. Children with severe TD sometimes experience physical pain, social isolation, and emotional disturbances, and they are at risk of performing poorly at school, which can seriously affect their quality of life. The age of onset of these disorders is usually before 18 years, and they most frequently occur 4 and 8 years, with an average age of 6 years. The most severe cases are seen between 10 and 12 years of age (1). The aetiology and pathogenesis of TD may be influenced by a variety of factors, such as genetics, neuroanatomy, neurobiology, immune dysfunction, psychology, and social environments (2–4). Currently, the most recognised mechanism underlying TD’s pathogenesis may be the disinhibition of the cortical–striatal–thalamic–cortical circuit. Disinhibition–excitation signals are unbalanced in this circuit, resulting in tics and their associated symptoms. For example, over-activity of dopamine in the striatum or over-sensitivity of post-synaptic dopamine receptors can lead to tic symptoms (5). Weakened activation signals of the anterior cingulate gyrus between key network nodes in the frontoparietal lobe and dysregulation of information flow lead to frequent involvement of neural pathways, thereby resulting in tics (6).

Vitamin D is a steroid hormone, derived by the synthesis of vitamin D3 from subcutaneous 7-dehydrocholesterol under sunlight or from vitamin D2 in food, both of which are metabolised into 25-hydroxyvitamin D in the liver. Vitamin D deficiency often occurs because of insufficient exposure to sunlight. Recently, it has been reported that vitamin D deficiency is associated with TD; children with TD had significantly greater vitamin D deficiency than healthy controls. Additionally, the levels of serum 25-hydroxyvitamin D in children with TD were significantly lower than those in healthy children (7–10). Previous studies have reported that the severity of TD is highest between 10 and 12 years of age and that symptoms of TD can be improved by vitamin D supplementation (1, 11). Contrastingly, some studies report no relation between vitamin D deficiency and severity of TD (12, 13). To verify the correlation between vitamin D levels and TD severity, we evaluated this association in children with TD and in healthy controls in Shanghai, China. Our research may provide new evidence for the prevention and treatment of TD.

## Materials and Methods

### Study Procedure

We retrospectively reviewed the electronic medical record database of paediatric patients diagnosed with TD at the Shanghai Children’s Hospital between May 1, 2019 and December 31, 2021. The participants were mainly patients with TD who first visited the outpatient department and had not received any drug treatment intervention. The inclusion criteria of the TD group were as follows: (1) diagnosis of TD following a comprehensive assessment according to the Diagnostic and Statistical Manual of Mental Disorders, Fifth Edition (DSM-5): one or more motor tics or vocal tics occurring several times daily; (2) age 5–14 years; (3) no history of TD treatment; and (4) possibility of other physical illnesses and substance use that can exhibit similar symptoms being completely ruled out. The exclusion criteria were as follows: (1) a neuroimaging-based diagnosis of brain structural abnormalities; (2) comorbidities such as cardiovascular diseases, severe malnutrition, and severe digestive system diseases; and (3) other serious medical diseases identified by a detailed examination. Healthy controls [children aged 5–14 years who had never been diagnosed with TD or any other neurodevelopmental disorders, such as obsessive–compulsive disorder, autism, or attention deficit hyperactivity disorder (ADHD)] underwent health check-ups during the same time period.

A questionnaire on family environmental factors was used to collect basic information on children in the TD group, including gender, age, number of family members, family history of TD, living environment (urban/suburban), daily outdoor activities, and more; the questionnaires were completed by children or their guardians. The quality inspector was assigned to conduct a review, and 10% of the questionnaires were randomly selected for a telephonic survey to verify the authenticity of the content. A total of 2960 and 2655 children were enrolled in the TD and control groups, respectively. Their mean ages were 7.71 ± 2.7 and 8.61 ± 2.5 years, respectively. For further analysis, the children with TD were classified into five subgroups, according to their age (5–6 years, *n* = 1052; 7–8 years, *n* = 909; 9–10 years, *n* = 656; 11–12 years, *n* = 279; 13–14 years, *n* = 64).

The Yale Global Tic Severity Score (YGTSS) is a clinician-rated instrument to assess the severity of tics. It consists of three domains: (1) identification of motor/vocal tic symptoms; (2) a score-system to assess the severity of motor and vocal tics separately across five dimensions, including quantity of tics and tic frequency, intensity, complexity, and interference with daily life over the prior week; and (3) a scale of functional impairments in self-esteem, social interaction, studying of children with TD. Each domain is scored on a 6-point scale (range 0–5), with a separate rating for “overall impairment” in the individual’s daily life (14). Five sum scores are then created: the total motor tic score (range 0–25), the total phonic tic score (range 0–25), the total tic score (TTS; sum of the total motor tic score and total phonic tic score), the overall impairment rating (one item; range 0–50), and the global severity score (sum of the TTS plus the overall impairment rating; range 0–100). TD cases with a total YGTSS score of less than 25 are considered mild; 25–50 as moderate; and greater than 50 as severe (15).

### 25-Hydroxyvitamin D Detection

Blood (3–5 ml) was collected intravenously from both groups in the morning while subjects remained in a fasting state. Serum was obtained after centrifugation of blood at 3000 rpm for 10 min. Serum levels of 25-hydroxyvitamin D were detected by an electrochemiluminescence method *via* a Roche Electrochemiluminescence Analyser Cobase E601 (Germany). The reagent for detection of 25-hydroxyvitamin D and calibration were provided by Roche Diagnostic Company, Germany. As defined by the American Academy of Pediatrics, a serum level of 25-hydroxyvitamin D ≤ 37.5 nmol/L was considered deficient; 37.5 nmol/L < 25-hydroxyvitamin D ≤ 50 nmol/L was considered insufficient; and 25-hydroxyvitamin D ≥ 50 nmol/L was considered sufficient (16).

### Statistical Analysis

SPSS 26.0 statistical software was used to analyse the data. Non-parametric Mann–Whitney *U* testing was used for the comparison of the quantitative data of the two grouping grades, and Kruskal–Wallis testing was used for the comparison of the counting data of three or more grouping grades, which are expressed as *n* (%). The Shapiro–Wilk test was used to test the normality of measurement data. Non-parametric Mann–Whitney *U* testing was used for comparison of binary measurement data that did not achieve normality. Kruskal–Wallis testing was used for the comparison of three or more groups, which are expressed in the M (P25, P75) form. The Bonferroni correction method was used for *post hoc* comparison and was written in letters. Receiver operating characteristic (ROC) curve analysis of vitamin D levels at different ages was performed on “differentiated” TD and on normal children to analyse the diagnostic power of vitamin D levels at different ages. The test level α was set to 0.05.

## Results

**FIGURE 1:**
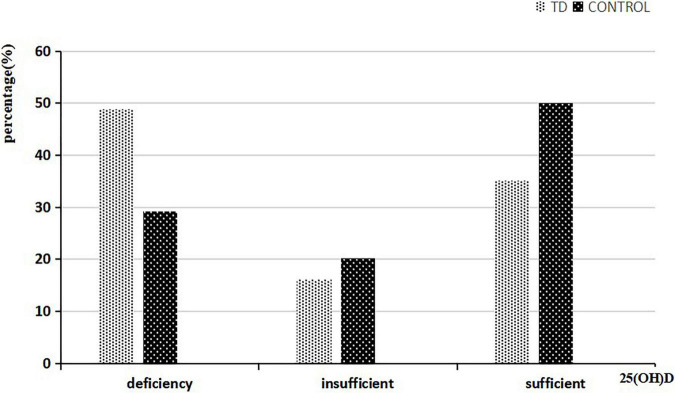
Comparison of the different levels of 25-hydroxyvitamin D in the two groups.

Of the 2665 children in the control group, 1912 were boys (71.7%) and 753 were girls (28.3%). Of the 2960 children in the TD group, 2355 were boys (79.6%) and 560 were girls (20.4%). The average level of 25-hydroxyvitamin D in the TD group was 38.47 (23.56–58.89) nmol/L, which was significantly lower than that in the control group, which was 50.05 (34.47–65.80) nmol/L (*Z* = −14.514, *P* < 0.001). Using the previously defined criteria, we found that the deficiency rate of 25-hydroxyvitamin D in the TD group was significantly higher than that in the control group (*Z* = −14.238, *P* < 0.001) (Figure 1).

There were no significant differences in living environments or 25-hydroxyvitamin D deficiency grades between boys and girls in the TD group; however, there was a significant difference in the time spent outdoors (*P* < 0.001; Table 1).

**TABLE 1:** Basic characteristics of the TD group.

Basic characteristics	Gender		*Z*/χ^2^	*P*
	Boy (*n* = 2355)	Girl (*n* = 605)		
Age (year)	8 (6.42, 9.75)	7.42 (5.92, 9.17)	−5.116	0.000
Urban (%)	1880 (79.83)	507 (83.80)	4.863	0.027
Suburban (%)	475 (20.17)	98 (16.20)		
Time spent outdoors <1 h (%)	1832 (77.79)	433 (71.24)	13.568	0.001
1–2 h (%)	398 (16.90)	141 (23.31)		
>2 h (%)	125 (5.31)	33 (5.45)		
25-Hydroxyvitamin D			−0.18658	0.853
Deficiency (%)	1155 (49.04)	290 (47.93)		
Insufficient (%)	370 (15.71)	105 (17.36)		
Sufficient (%)	830 (35.24)	210 (34.71)		

According to the Kruskal–Wallis test, there were no significant differences in YGTSS score grades when comparing 25-hydroxyvitamin D levels and degrees of 25-hydroxyvitamin D deficiency in the TD group (*P* > 0.05) (Table 2).

**TABLE 2:** Receiver operating characteristic curve analysis of 25-hydroxyvitamin D levels in different age samples of two groups.

Age	AUC	*P*	95% CI	Cut-off	Sensitivity %	Specificity %
Total	0.612	<0.001	0.597–0.627	35.34	46.28	74.11
5–6 years	0.610	<0.001	0.584–0.636	38.81	44.51	75.59
7–8 years	0.621	<0.001	0.595–0.648	33.29	43.78	78.55
9–10 years	0.622	<0.001	0.591–0.653	34.84	52.28	71.00
11–12 years	0.642	<0.001	0.597–0.686	30.43	46.60	76.78
13–14 years	0.640	0.001	0.557–0.723	19.86	35.94	91.86

**FIGURE 2:**
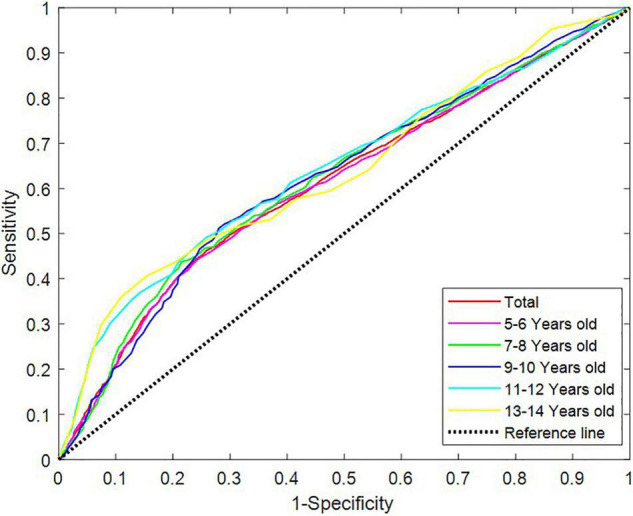
Receiver operating characteristic curves of vitamin D levels in different age groups. ROC, receiver operating characteristic.

Receiver operating characteristic curve testing was performed to analyse the 25-hydroxyvitamin D levels of all samples to “distinguish” the differences in characteristics between children with TD and healthy children. The critical level of 25-hydroxyvitamin D was determined to be 35.34 nmol/L, with a sensitivity of 46.28% and specificity of 74.11%, indicating a low diagnostic efficacy (Table 3). In different age groups, the area under the curve (AUC) of the ROC curve of 25-hydroxyvitamin D ranged from 0.610 to 0.642 (*P* < 0.05). Although the level of 25-hydroxyvitamin D in the same age group could distinguish children with TD from healthy children, the diagnostic efficacy was still low (Figure 2).

**TABLE 3:** Comparison of YGTSS score and 25-hydroxyvitamin D levels in the TD group.

YGTSS	Number	25-Hydroxyvitamin D	25-Hydroxyvitamin D		
			Deficiency	Insufficient	Sufficient
Mild	2131	38.94 (23.68, 59.29)	1038 (48.71)	344 (16.14)	749 (35.15)
Moderate	692	37.94 (23.22, 58.71)	340 (49.13)	102 (14.74)	250 (36.13)
Severe	137	38.04 (23.52, 56.05)	67 (48.91)	29 (21.17)	41 (29.93)
*H*		1.860	0.391		
*P*		0.395	0.822		

Mean levels of 25-hydroxyvitamin D in children of different ages were compared, and the differences were statistically significant (*P* < 0.001). The average level of 25-hydroxyvitamin D in children aged 5–6 years and 7–8 years was higher than that in children over 9 years old (*P* < 0.05). Non-parametric Kruskal–Wallis testing showed significant differences in vitamin D deficiency in children of different ages, especially in adolescents over 9 years of age (*P* < 0.001) (Table 4).

**TABLE 4:** Comparison of vitamin D levels and degree of deficiency across different age groups in the TD group.

Age	Number	25-Hydroxyvitamin D	25-Hydroxyvitamin D		
			Deficiency	Insufficient	Sufficient
5–6 years	1052	44.14 (26.07, 64.99)a	451 (42.87)	153 (14.54)	448 (42.59)
7–8 years	909	37.85 (24.18, 57.50)b	450 (49.50)	145 (15.95)	314 (34.54)
9–10 years	656	33.51 (21.77, 53.37)c	356 (54.27)	110 (16.77)	190 (28.96)
11–12 years	279	33.01 (19.52, 50.16)c	153 (54.84)	56 (20.07)	70 (25.09)
13–14 years	64	30.59 (16.65, 52.02)c	35 (54.69)	11 (17.19)	18 (28.13)
*H*		83.752	42.374		
*P*		<0.001	<0.001		

*Annotation (a, b, c): different letters indicated statistically significant difference between groups (P < 0.05).*

## Discussion

### Main Findings

This research analysed 25-hydroxyvitamin D levels in children with TD in Shanghai and explored the characteristic differences between them and healthy children. To increase the reliability of our results, we ensured that the sampling time frame spanned a period of over 2 years; the time of year remained relatively uniform across instances of sampling, and the sample size was large to offset the effect of missing data. Our results showed that the average level of 25-hydroxyvitamin D in the TD group was significantly lower than that in healthy children, and the proportion of 25-hydroxyvitamin D deficiency in the TD group was significantly higher than that in healthy children. However, there was no correlation between 25-hydroxyvitamin D deficiency and the severity of TD. Regarding the family questionnaire findings in the TD group, we found that TD was more common in boys. The ratio of male to female was 3.89:1, and boys were slightly older than girls on average. TD in Shanghai appears to be related to the daily time spent performing outdoor activities. Approximately 80% of the children with TD in the sample were from urban areas, and 76.5% of the children answered that they spent less than 1 h performing outdoor activities every day. This may be related to local influences; Shanghai is a modern city with a dense population, which may be associated with increased pressure on students to perform well academically, a higher number of dual-income parents, and less time and venues for children to perform outdoor activities; children are thus mainly exposed to indoor rather than outdoor training, education, and amusement. This in turn leads to reduced exposure to sunlight and insufficient vitamin D production all year round. Parents also have an insufficient awareness of routine vitamin D supplementation for children. Although the Endocrine Society of America has recommendations in place to prevent nutritional rickets, daily doses of 400–1000 IU in infants, 600–1000 IU in children, and 1500–2000 IU in teenagers are needed (17). In China, vitamin D deficiency and rickets prevention and treatment recommendations have been issued twice, but vitamin D supplementation is still mainly only undertaken for children under the age of 3 years (18). The vitamin D supplementation rate in children aged 3–12 years was reported to be only 16.6%, and it decreased significantly with an increase in age (19). Because for serum vitamin D levels depend on dietary intake (20%) and sun exposure (80%), lack of supplementation combined with insufficient sunlight can lead to vitamin D deficiency. Furthermore, in our study, vitamin D deficiency did not appear only in patients with TD, but also in healthy children (insufficiency: 49.9%). In children with TD, vitamin D deficiency is more pronounced than in those without tics. ROC curve revealed that 25-hydroxyvitamin D levels have a low diagnostic efficacy in distinguishing children with TD from healthy children (Table 2). Whether children with TD are more prone to vitamin D deficiency and whether those with 25-hydroxyvitamin D deficiency are more likely to develop TD, requires further investigation.

We studied the relation between 25-hydroxyvitamin D levels and its degree of deficiency in children of different ages in the TD group. The 25-hydroxyvitamin D levels of children of different ages varied, and the 25-hydroxyvitamin D levels of children over 9 years old were significantly lower than those of children of all other age groups. Children aged 5–8 years primarily had insufficient levels, and those aged over 9 years primarily had significant deficiencies (Table 4). ROC analysis was performed on the levels of 25-hydroxyvitamin D in different age groups. The AUC of the ROC curve of 25-hydroxyvitamin D ranged from 0.610 to 0.642 (*P* < 0.05). Although the level of 25-hydroxyvitamin D within the same age group helped distinguish children with TD from healthy children, the diagnostic efficacy was still low (Figure 2).

### Status of 25-Hydroxyvitamin D

Vitamin D is a fat-soluble vitamin that is naturally present in some foods and is mainly produced in the skin when it is exposed to sunlight (UVB radiation). Its synthesis is therefore greatly influenced by the season, latitude, air pollution, skin pigmentation, sunscreen use, and ageing, as well as by genetic factors, altered absorption, metabolism, and medication (12, 20). Serum 25-hydroxyvitamin D is the best indicator of vitamin D status. Approximately 40% of the world’s population is vitamin D deficient, with 25-hydroxyvitamin D levels <50 nmol/L, and 60% of the world population has insufficient vitamin D levels (50–79 nmol/L) (21). As per recommendations made by the European Society of Gastroenterology, Hepatology, and Nutrition, 25-hydroxyvitamin D levels <25 nmol/L are considered deficient, and levels >50 nmol/L are considered sufficient (22); substantially higher levels of >75 nmol/L were recommended as sufficient by the United States Endocrine Society. This threshold was based on a suggested plateau in parahormone status, as well as on an observed optimum for non-vertebral fracture prevention and intestinal calcium absorption (17, 23). In 2015 the Research Cooperation Group of Rickets Prevention and Treatment in China and the Professional Committee of Pediatric Nutrition of the Chinese Healthy Birth Science Association suggested that a serum vitamin D level reaching 50–250 nmol/L is appropriate for children in China (24). Research reports that achieving circulating concentrations of 25-hydroxyvitamin D in the range of 100–150 nmol/L appears to optimise vitamin D’s effect on improving immune function, thereby substantially reducing the risk for serious infections, particularly from severe acute respiratory syndrome coronavirus 2; the hypothesised mechanism involves modulation of the immune response, helping prevent a dangerous and often fatal cytokine storm (21). Despite the ubiquitous modern way of life that limits opportunities for sunlight exposure, we suggest that people increase their time spent outside; we emphasise this especially for children and teenagers, and we recommend strengthening support for food rich in 25-hydroxyvitamin D. Moreover, additional vitamin D supplements are often needed to ensure recommended 25-hydroxyvitamin D levels in the body. In 2011, the Endocrine Society’s guidelines committee suggested daily doses of 400–1000 IU in infants, 600–1000 in children, and 1500–2000 in teenagers to prevent nutritional rickets (17).

### 25-Hydroxyvitamin D and Tic Disorders

In addition to its role in the regulation of calcium and phosphorus metabolism and in the promotion of bone health, 25-hydroxyvitamin D plays an important role in the development and function of the nervous system, such as mediating anti-trophic nerve cytokines, regulating cell proliferation and differentiation, promoting neuronal development, and regulating the synthesis of neurotransmitters (25–27). 25-Hydroxyvitamin D directly regulates the expression of the dopamine-rate-producing enzyme tyrosine hydroxylase, which is responsible for the conversion of the amino acid L-tyrosine, a precursor of dopamine, into dopamine, which in turn is also a precursor of adrenaline and norepinephrine. However, vitamin D receptors and vitamin D-related metabolic enzyme 1-α hydroxylase are widely distributed in dopamine-rich regions, such as the substantia nigra and striatum. Therefore, when the serum 25-hydroxyvitamin D level is deficient in a pathological state, the expression of tyrosine hydroxylase is insufficient, which promotes the release of dopamine in the striatum. The cortical–striatal–thalamic–cortical circuits are then disinhibited, and disinhibition–excitation signals are unbalanced in these circuits, resulting in tics and related symptoms (5, 28, 29). Millet et al. argue that 25-hydroxyvitamin D deficiency is a biological risk factor for neuropsychiatric diseases, which may be associated with autism, ADHD, dementia, cognitive impairment, Alzheimer’s disease, Parkinson’s disease, depression, schizophrenia, and other diseases (30).

Tic disorders are common childhood-onset neurological diseases. However, the pathophysiology underlying these disorders is unclear, except for the current focus on the disinhibition of the corticostriatal–thalamocortical circuit. Autoimmune dysfunction has been proposed in the pathogenetic mechanism of TD and related neuropsychiatric disorders, such as obsessive–compulsive disorder and ADHD (31). The release of both innate and adaptive immune cells is modulated by effector molecules, including cytokines, chemokines, and adhesive molecules. Leckman (32) reported increased baseline plasma levels of the proinflammatory cytokines tumour necrosis factor α (TNF-α) and IL-12 in children with TD and/or early-onset obsessive–compulsive disorder. Levels of these two cytokines further increased during periods of symptom exacerbation. A later study also showed elevated levels of proinflammatory cytokines, including IL-12 and TNF-α, in paediatric patients with TD and without obsessive–compulsive disorder. Additionally, children who are medication-naïve showed higher TNFα levels than healthy controls (33). Taken together, the proinflammatory immune response may be at least partly related to the pathogenesis of TD (3). 25-Hydroxyvitamin D affects the development of TDs through its regulatory role in innate and adaptive immunity. The vitamin D receptor (VDR) is widely distributed in various tissues of the body, such as immune cells and nerve tissues. Vitamin D binds to VDR, and it can inhibit the release of proinflammatory cytokines, such as interleukin (IL)-12, IL-2, cell interferon-γ, and TNF-α, as mediated by helper T cells; vitamin D can also induce the expression of Th2 cells and regulatory T cells, thereby increasing the synthesis and release of the anti-inflammatory cytokines IL-3, IL-4, and IL-10 (34). Thus, 25-hydroxyvitamin D deficiency leads to elevated baseline plasma levels of the proinflammatory cytokines TNF-α and IL-12, leading to tic symptoms.

25-Hydroxyvitamin D signal transduction enhances innate immunity for pathogens of bacterial or viral origin and inhibits inflammatory immune responses, which are the basis of autoimmunity (35). Evidence suggests that a clear association exists between anti-streptococcal antibody titres and TDs, further strengthening the relationship between tics and streptococcal infections (36). 25-Hydroxyvitamin D deficiency may also impair innate and acquired immune responses, leading to increased vulnerability to infection responses and disease (37). Improved 25-hydroxyvitamin D status has a significant effect on immune cell activity, and several studies have demonstrated that improved 25-hydroxyvitamin D status reduces the risk of upper respiratory tract viral infections and causes a variety of innate and acquired immune effects. In particular, circulating concentrations of 25-hydroxyvitamin D in the 100–150 nmol/L range seem to optimise the effect of vitamin D on improving immune function, thus greatly reducing the risk of infections (21, 38).

There may also be a genetic component to 25-hydroxyvitamin D deficiency in TDs. Vitamin D can regulate metabolism through its own genes or VDR genes. Serum 25-hydroxyvitamin D levels are influenced not only by exposure to ultraviolet light, but also by age, body mass index, skin colour, and numerous other factors regulating exposure to ultraviolet B radiation (including season, geographical latitude, and skin coverage). In addition, 50–80% of the variability in the concentration of 25-hydroxyvitamin D is explained by genetic factors. Studies have found that 25-hydroxyvitamin D genes are mostly enriched in liver and lipid metabolism gene pathways and are enriched and expressed in liver, skin, and gastrointestinal tissues (39). In addition, the VDR gene is located on chromosome 12 (12q13.11), and more than 900 allelic variants in the VDR locus have been reported. The VDR is a member of the nuclear receptor superfamily of transcriptional regulators, playing a crucial role in calcitriol or 1-alfa,25-dihydroxycholecalciferol (1α,25(OH)2D) signalling. The VDR is activated by binding with 1α,25(OH)2D, which forms a heterodimer with the retinoid X receptor (RXR). The 1α,25(OH)2D-VDR-RXR complex migrates to the nucleus to regulate the transcription of genes involved in vitamin D effects, including phosphorous and calcium metabolism, cell proliferation, and the control of innate and adaptive immunity. The best studied VDR gene polymorphisms are Apal (rs7975232), *Bsm*I (rs1544410), Taql (rs731236), and Fokl (rs10735810). These genetic variants have been associated with a predisposition to chronic diseases such as autoimmune diseases, rheumatic arthritis, and metabolic bone diseases (40–42).

25-Hydroxyvitamin D plays an important role during pregnancy; animal experiments have shown not only that 25-hydroxyvitamin D deficiency can affect the normal development and function of the dopamine system during embryonic development, but also that the effects of 25-hydroxyvitamin D deficiency on neurotransmitters in the brain are likely to be maintained for a long time and even extend to the offspring (43). Higher prenatal circulating 25-hydroxyvitamin D concentrations may have positive effects on neurodevelopment in offspring, including improved cognitive development and a reduced risk of ADHD and autism-related traits (44). Higher levels of maternal 25-hydroxyvitamin D in the third trimester of pregnancy are also associated with a lower risk of ADHD in offspring (45). An adequate supply of vitamin D during pregnancy can improve maternal and foetal outcomes and the short- and long-term health of the offspring (46). Therefore, we speculate that children with TD may have low levels of 25-hydroxyvitamin D during the intrauterine period or postnatal infancy. However, this claim lacks evidence from longitudinal observational studies.

### Limitations, Conclusion, and Future Implications

We performed a retrospective study elucidating the level of 25-hydroxyvitamin D, severity of TD, living environment, and other factors of outpatient children with TD. To the best of our knowledge, this is the first large-scale study evaluating all of these factors at once. Restricted by research conditions, patients were selected according to the diagnostic and exclusion criteria of TD, and there was no systematic investigation on the complications of TD, particularly those involving or related to mental and psychological diseases. Therefore, our research lacks relevant data on the correlation between vitamin D deficiency and TD complications. In addition, we hope that future studies on vitamin D supplementation will make up for the lack of longitudinal data on treatment follow-up for TD in this project.

In conclusion, our study confirms that 25-hydroxyvitamin D deficiency is prevalent in children with TD, but the degree of that deficiency is not significantly correlated with the severity of TD. Levels of 25-hydroxyvitamin D deficiency correlated with age, especially in children over 9 years of age. In the future, we plan to select appropriate treatment objectives and conduct vitamin D supplementation therapy for TD patients with 25-hydroxyvitamin D deficiency according to international vitamin D supplement treatment standards, to further verify the clinical therapeutic effects of vitamin D.

## Data Availability Statement

The original contributions presented in the study are included in the article/supplementary material, further inquiries can be directed to the corresponding author.

## Ethics Statement

The research protocol for this study was approved by the Institutional Review Committee of Shanghai Children’s Hospital. Owing to the retrospective study design, the requirement for informed consent was waived by the Institutional Review Board.

## Author Contributions

YC: conceptualisation and supervision. QX and XL: data curation. FY: funding acquisition. YR and XJ: investigation. YZ: project administration. AW: software. SW and YW: writing – original draft. MG and WZ: writing – review and editing. All authors have read and approved the final manuscript.

## Conflict of Interest

The authors declare that the research was conducted in the absence of any commercial or financial relationships that could be construed as a potential conflict of interest.

## Publisher’s Note

All claims expressed in this article are solely those of the authors and do not necessarily represent those of their affiliated organizations, or those of the publisher, the editors and the reviewers. Any product that may be evaluated in this article, or claim that may be made by its manufacturer, is not guaranteed or endorsed by the publisher.
